# Caspase-1 and Inflammasome Signaling in Spinal Cord Injury: Mechanistic Insights and Translational Gaps

**DOI:** 10.3390/ijms27198802

**Published:** 2026-10-01

**Authors:** Adonicah Y. Cummings, Adham M. Khalafallah, Bhavjeet S. Sanghera, Isabella R. Chamma, Emily L. Errante, Adam S. Levy, Hisham F. Bahmad, W. Dalton Dietrich, Juan Pablo de Rivero Vaccari, S. Shelby Burks

**Affiliations:** 1 Herbert Wertheim College of Medicine, Florida International University, Miami, FL 33199, USA; acumm012@fiu.edu; 2 Department of Neurological Surgery, University of Miami Miller School of Medicine, Miami, FL 33136, USA; akhalafallah@med.miami.edu (A.M.K.); bss155@med.miami.edu (B.S.S.); ele28@med.miami.edu (E.L.E.); adam.levy@med.miami.edu (A.S.L.); ddietrich@med.miami.edu (W.D.D.); jderivero@med.miami.edu (J.P.d.R.V.); sburks@med.miami.edu (S.S.B.); 3 Department of Neurological Surgery, Rutgers New Jersey Medical School, Newark, NJ 07103, USA; ic404@njms.rutgers.edu; 4 The Miami Project to Cure Paralysis, University of Miami Miller School of Medicine, Miami, FL 33136, USA; 5 Department of Pathology and Laboratory Medicine, University of Miami Miller School of Medicine, Miami, FL 33136, USA

**Keywords:** caspase-1, inflammasome, pyroptosis, spinal cord, biomarkers

## Abstract

Caspase-1 is a central mediator of canonical inflammasome signaling and drives neuroinflammatory injury through interleukin-1β (IL-1β) and interleukin-18 (IL-18) maturation, gasdermin D (GSDMD)-mediated pyroptosis, and secondary inflammatory cascades. In spinal cord injury (SCI) and other neurologic disorders, experimental evidence links caspase-1 activation to blood–spinal cord barrier (BSCB) or blood–brain barrier (BBB) disruption, glial activation, neuronal and oligodendroglial injury, and worse functional outcomes. However, despite mechanistic rationale, the clinical role of caspase-1 as a biofluid biomarker remains incompletely defined. This review summarizes preclinical and clinical evidence regarding caspase-1 and inflammasome-associated proteins across cerebrospinal fluid (CSF), serum, plasma, and other biofluids, emphasizing SCI and translational neuroinflammation. Human studies suggest that inflammasome-associated markers may reflect acute inflammatory activity and injury severity, but direct caspase-1 measurement is limited by small cohorts, heterogeneous sampling windows, variable assay platforms, and inconsistent correlations with neurologic outcomes. Overall, caspase-1 remains a biologically plausible but not clinically established biomarker. Future studies should incorporate longitudinal sampling, standardized assays, paired measurement of IL-1β, IL-18, GSDMD, and other pyroptosis-related markers, and integration with imaging and functional outcomes. Such work may clarify whether caspase-1 can serve as a clinically actionable biomarker, prognostic indicator, or therapeutic monitoring target in SCI and related neuroinflammatory conditions.

## 1. Introduction

Spinal cord injury (SCI) remains among the most devastating neurologic insults, with an incidence of approximately 18,000 new cases per year and a prevalence of about 255,000 to 390,000 individuals in the United States [1]. Despite early intervention, management of SCI has historically been difficult, with many patients experiencing permanent neurologic deficits, often requiring significant functional assistance and/or full-time care [2]. The long-term consequences of SCI carry significant financial burden at both the individual and systemic level, with estimated lifetime costs ranging from $2 million to over $6 million [1].

SCI involves a complex pathophysiological cascade that begins at the moment of trauma, and continues with lingering secondary effects mediated by a robust inflammatory response [3,4]. This process begins with direct mechanical compression (primary injury). Furthermore, impingement of the spinal cord results in contusion of the neural tissue with or without damage to the surrounding macrovascular supply (i.e., the anterior or posterior spinal arteries) [5]. In the minutes following the initial mechanical destruction of neural tissue, one enters the secondary phase of injury, marked by a complex cascade of biochemical and cellular processes that amplify tissue damage and drive lesion progression [3]. This secondary injury cascade is the primary target of therapeutic interventions and ongoing research, encompassing processes such as inflammation, oxidative stress, blood–spinal cord barrier (BSCB) disruption, and programmed cell death [6].

Among these secondary injury cascade processes, activation of the canonical inflammasome signaling pathway has emerged as a central driver of post-traumatic neuroinflammation. Caspase-1 regulates the maturation of interleukin (IL)-1β and IL-18, and induces pyroptotic cell death by cleaving gasdermin-D (GSDMD), thus linking inflammatory signaling to structural tissue damage and cell death [7,8,9]. Robust preclinical studies have demonstrated early inflammasome activation in experimental SCI, and pharmacologic inhibition of caspase-1 has been shown to improve histopathologic and functional outcomes in rodent models [10,11,12,13,14,15,16].

Despite extensive preclinical evidence implicating caspase-1 in SCI, its evaluation as a measurable biofluid biomarker in human SCI has remained limited [17]. Consequently, its potential utility for assessing inflammasome activation, stratifying patients, and monitoring responses to inflammasome-targeted therapies remains uncertain.

This review synthesizes current evidence supporting caspase-1 activation in SCI, evaluates existing human data, and outlines a translational framework for developing inflammasome-derived biofluid biomarkers for acute SCI.

## 2. Introduction to Secondary Injury

The secondary injury phase begins within minutes of the initial mechanical insult and evolves over hours to days, driven by biochemical and cellular cascades that expand the initial lesion [18]. Secondary injury progresses through temporally defined stages, commonly categorized as acute, subacute, and chronic [4]. The acute phase marks the onset of these secondary events, including oxidative stress, early inflammatory signaling, glial activation, and BSCB breakdown [19].

### 2.1. Oxidative Stress

During this early period, oxidative stress rapidly becomes a major amplifier of secondary injury, in part because the central nervous system’s high lipid content makes it an ideal environment for oxidative stress signaling. Normally, the central nervous system has multiple endogenous antioxidants that maintain redox balance [20]. However, the abrupt surge in reactive oxygen species (ROS) after SCI quickly overwhelms these systems. Excess free radicals drive lipid peroxidation, disrupt membrane stability, impair sodium-potassium ATPase activity, and trigger a variety of programmed and non-programmed cell death processes [20,21,22]. Moreover, redox-mediated processes magnify cellular dysfunction and create a permissive environment for the subsequent inflammatory, glial, and vascular responses that characterize the evolving secondary injury cascade.

### 2.2. Inflammation

As the injured microenvironment becomes destabilized in the immediate aftermath of SCI, multiple danger signals, including oxidative stress, ionic imbalance, and cellular debris, collectively trigger a robust inflammatory response [23]. Although inflammation ordinarily contributes to tissue repair, the acute post-injury response in SCI is disproportionate and quickly adopts a pathologic role, thereby exacerbating ongoing secondary injury. Resident microglia rapidly release pro-inflammatory cytokines such as tumor necrosis factor (TNF), IL-1β, and IL-6, while circulating neutrophils infiltrate the lesion and amplify oxidative and enzymatic injury [24,25,26]. Furthermore, activated microglia and macrophages then sustain this heightened inflammatory state over hours to days, hence exacerbating neuronal and glial dysfunction while setting the stage for subsequent cytokine waves, glial reactivity, and vascular compromise [25,27,28].

### 2.3. Glial Activation

Beyond their involvement in the inflammatory cascade, microglia and infiltrating macrophages undergo dynamic phenotypic changes after SCI that strongly influence the injured tissue. Following injury, macrophages accumulate within the lesion core, while resident microglia predominate in the perilesional region. Together, they peak around 7–10 days before gradually declining in the subacute phase [29]. These cells rapidly adopt predominantly pro-inflammatory, M1-like phenotypes but retain the capacity to shift toward more reparative, M2-like phenotypes depending on microenvironmental cues [30]. Activated microglia release IL-1α, TNF, and C1q, inducing the transition of resting astrocytes into A1 reactive astrocytes [31]. Once formed, A1 astrocytes exhibit a neurotoxic phenotype characterized by loss of homeostatic functions and promotion of neuronal and oligodendrocyte injury [27]. In parallel, pro-inflammatory microglia secrete mediators such as TNF, nitric oxide, and complement that induce oligodendrocyte and oligodendrocyte progenitor cell death, as well as impaired differentiation and exacerbated demyelination [27,32]. Taken together, these evolving glial responses profoundly influence chronic tissue remodeling and functional recovery after SCI.

### 2.4. Blood–Spinal Cord Barrier (BSCB) Breakdown

**Figure 1:**
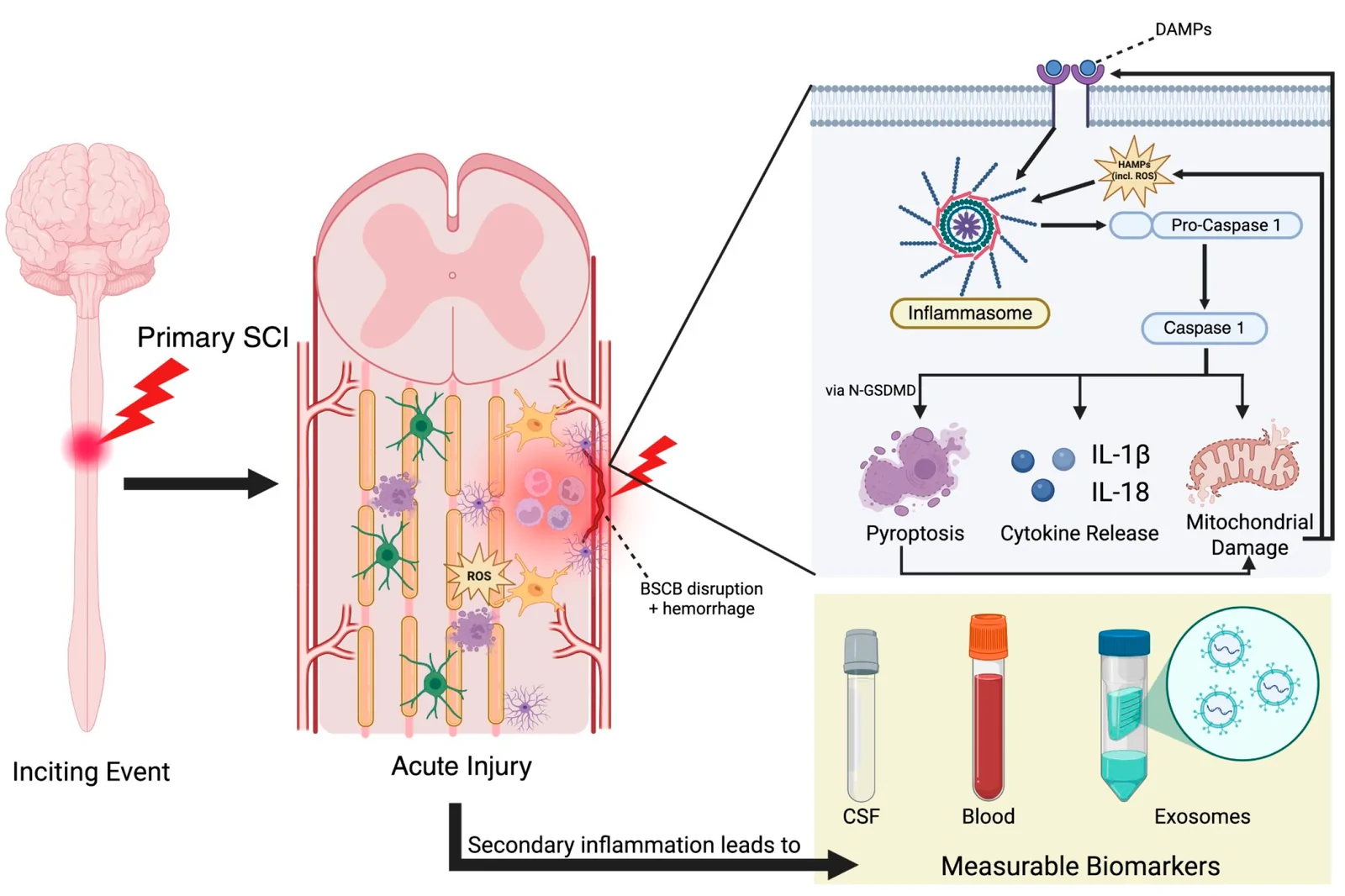
Inflammasome activation and potential biofluid biomarkers following acute spinal cord injury. Primary spinal cord injury (SCI) disrupts the blood–spinal cord barrier (BSCB), causes hemorrhage and oxidative stress, and releases damage-associated molecular patterns (DAMPs). These signals, together with homeostasis-altering molecular processes (HAMPs), including ionic disturbances and reactive oxygen species (ROS), promote canonical inflammasome assembly and caspase-1 activation, gasdermin D N-terminal fragment (GSDMD-N)-mediated pyroptosis, interleukin (IL)-1β and IL-18 release, and mitochondrial injury. Damaged mitochondria generate additional ROS and other HAMPs that feed back to amplify inflammasome activation. The resulting secondary inflammatory response may generate measurable biomarkers in cerebrospinal fluid (CSF), blood, and extracellular vesicles such as exosomes. The schematic illustration was created in Created in BioRender. Errante, E. (2026) https://BioRender.com/jx9gpys.

BSCB disruption is an early and critical component of secondary injury after SCI. Experimental studies show that BSCB permeability increases within hours of injury, peaking at approximately 24 h after SCI, and remains disrupted for several days before gradually normalizing by 10–14 days [33]. Mechanical trauma rapidly compromises endothelial integrity, leading to tight junction loss, increased permeability, and extravasation of plasma proteins and immune cells into the parenchyma [34,35]. This breakdown is further exacerbated by inflammatory mediators such as TNF, IL-1β, and matrix metalloproteinases [25,36]. The resulting vasogenic edema, ionic imbalance, and hemorrhage amplify neuronal and glial injury while facilitating sustained immune infiltration into the lesion core [37]. Moreover, persistent BSCB dysfunction in the subacute and chronic phases contributes to ongoing inflammation, impaired remyelination, and long-term neurodegeneration [28,34]. The relationship between these secondary injury processes, inflammasome activation, and the emergence of measurable biofluid biomarkers is summarized in Figure 1.

## 3. Caspase-1 and the Canonical Inflammasome Pathway

### 3.1. Canonical Inflammasome Assembly and Caspase-1 Activation

Inflammasomes are cytosolic signaling complexes that initiate the activation of pro-inflammatory cysteine aspartases, or caspases, most notably caspase-1, as part of the canonical inflammasome signaling pathway [38]. The inflammasome is composed of 3 key components: a sensor protein that detects cellular stress, such as NOD-like receptors (NLRP1, NLRP3) or AIM-like receptors (AIM2), an adaptor molecule known as apoptosis-associated speck-like protein containing a caspase recruitment domain (ASC), and an effector zymogen, caspase-1 [38,39,40,41].

In SCI, the NLRP3 sensor is the most extensively implicated inflammasome component and is expressed in neurons, microglia, and astrocytes [38]. Its activation is driven by injury-derived danger signals released after trauma—including extracellular ATP-induced potassium ion disturbances, mitochondrial dysfunction, and ROS formation [40,42]. Inflammasome assembly induces autocatalytic caspase-1 activation, which cleaves the inactive precursors pro-IL-1β and pro-IL-18 into mature, biologically active inflammatory cytokines [32]. Simultaneously, caspase-1 cleaves GSDMD, generating an N-terminal fragment that forms membrane pores through which IL-1β and IL-18 are released, ultimately contributing to cell swelling, rupture, and amplification of secondary injury [43]. This process of lytic, inflammatory cell death is referred to as pyroptosis [40,44]. However, this is not an all-or-none phenomenon.

### 3.2. Regulation of Pyroptotic Membrane Permeabilization and Rupture

Ninjurin-1 (NINJ1) has recently been identified as a critical mediator of plasma-membrane rupture (PMR), representing a terminal step in lytic cell death pathways, including pyroptosis [45]. Following inflammasome activation, caspase-1-mediated cleavage of gasdermin D (GSDMD) results in pore formation and initial membrane permeabilization [9,43]. However, pore formation alone is insufficient to induce complete cell lysis. Instead, NINJ1 is required for terminal membrane rupture and the release of intracellular damage-associated molecular patterns (DAMPs), thereby amplifying inflammatory signaling [45]. In Ninj1−/− models, cells undergo gasdermin-dependent pore formation and cytokine release but fail to undergo terminal membrane rupture, resulting in impaired release of HMGB1 and lactate dehydrogenase (LDH). These findings demonstrate that plasma membrane rupture is an actively regulated process rather than a passive consequence of osmotic lysis [45].

In contrast, not all GSDMD-mediated membrane damage culminates in cell lysis. Cells possess endogenous repair mechanisms, most notably the endosomal sorting complexes required for transport (ESCRT) machinery, which preserves membrane integrity following injury. ESCRT components are recruited to sites of membrane disruption, where they facilitate repair and removal of gasdermin-induced pores through ectosome shedding [46]. Notably, ESCRT depletion enhances pyroptotic cell death and interleukin-1β release without affecting ASC speck formation, caspase-1 activation, or GSDMD processing, indicating that ESCRT functions downstream of inflammasome signaling [46]. This repair response allows cells to transiently maintain membrane integrity, limiting full lysis while permitting controlled cytokine release. Collectively, these findings support a model in which ESCRT-mediated repair counteracts membrane damage, whereas NINJ1 drives irreversible plasma-membrane rupture [45,46].

**Figure 2:**
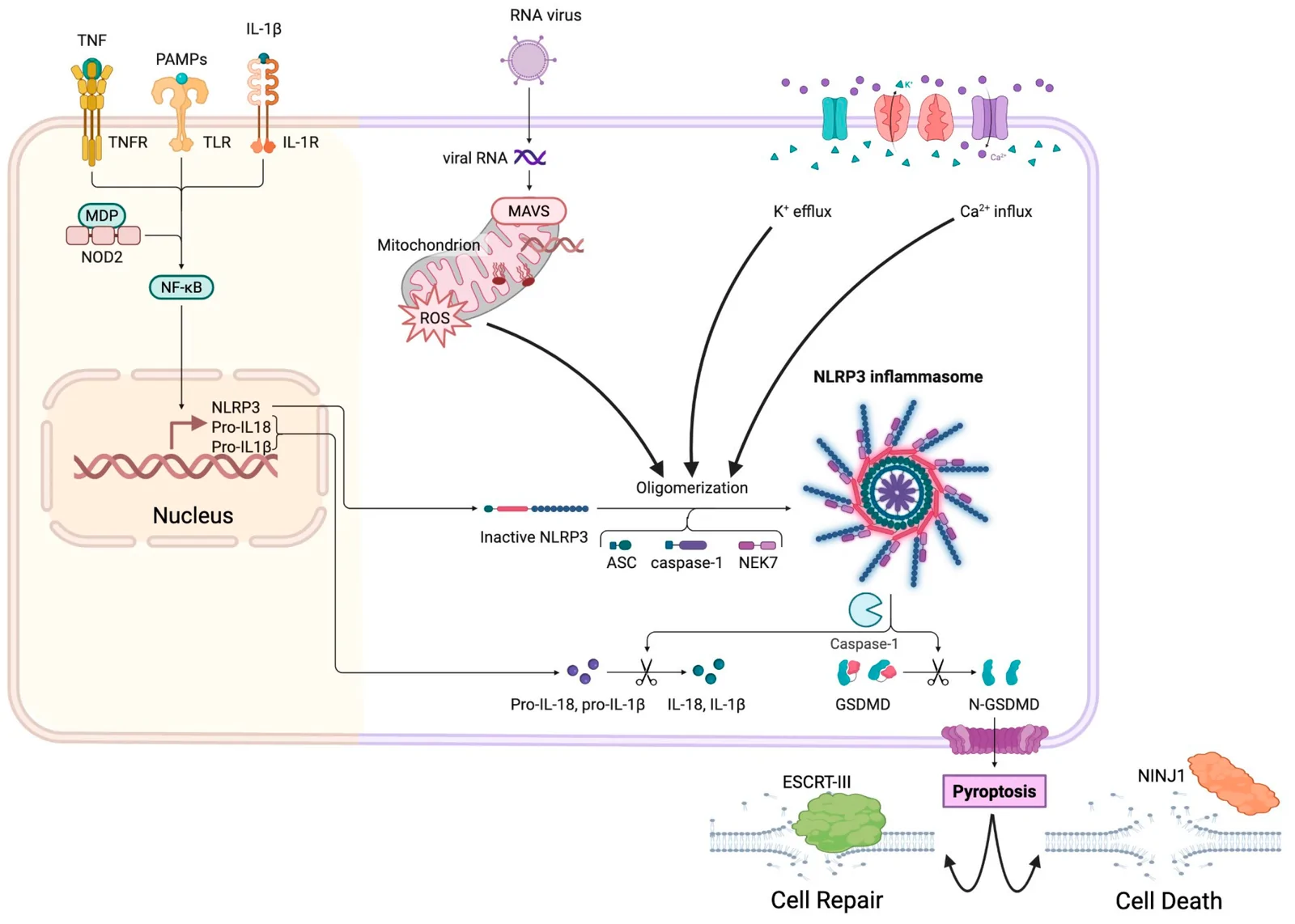
**Canonical NLRP3 inflammasome activation and regulation of pyroptotic cell death.** Priming signals initiated by tumor necrosis factor (TNF), pathogen-associated molecular patterns (PAMPs), interleukin-1β (IL-1β), or muramyl dipeptide (MDP) activate NF-κB through their respective receptors, inducing transcription of NLRP3, pro-IL-1β, and pro-IL-18. Subsequent activation signals, including mitochondrial reactive oxygen species (ROS), viral RNA–MAVS signaling, K^+^ efflux, and Ca^2+^ influx, promote NLRP3 oligomerization and recruitment of NEK7, ASC, and pro-caspase-1. Activated caspase-1 cleaves pro-IL-1β and pro-IL-18 into their mature forms and cleaves gasdermin D (GSDMD), generating pore-forming N-terminal GSDMD (N-GSDMD). GSDMD pore formation promotes cytokine release and pyroptosis. The ultimate cellular response is modulated by opposing membrane-remodeling pathways: ESCRT-III repairs GSDMD-mediated membrane injury and supports cell survival, whereas NINJ1 facilitates terminal plasma-membrane rupture and cell death. The schematic illustration was created in Created in BioRender. Chamma, I. (2026) https://BioRender.com/1vmtpl9. Abbreviations: ASC, apoptosis-associated speck-like protein containing a CARD; ESCRT-III, endosomal sorting complex required for transport III; IL-1R, interleukin-1 receptor; MAVS, mitochondrial antiviral-signaling protein; NEK7, NIMA-related kinase 7; NINJ1, nerve injury-induced protein 1; NOD2, nucleotide-binding oligomerization domain-containing protein 2; TLR, Toll-like receptor; TNFR, tumor necrosis factor receptor.

This balance between ESCRT-mediated repair and NINJ1-dependent membrane rupture has important implications for biomarker development. Sublytic pore formation permits the release of processed cytokines such as IL-1β and IL-18 without complete cellular disruption, whereas NINJ1-mediated PMR results in the liberation of intracellular contents, including DAMPs such as HMGB1 and cytosolic enzymes like LDH [45,46,47]. Thus, the relative contribution of membrane repair versus rupture may directly influence the composition and magnitude of biofluid biomarkers detected after spinal cord injury. Canonical NLRP3 inflammasome activation and the opposing roles of ESCRT-mediated membrane repair and NINJ1-dependent plasma-membrane rupture are summarized in Figure 2.

This mechanistic framework suggests a testable hypothesis for biomarker selection and interpretation. GSDMD pores can permit IL-1β secretion from viable macrophages before terminal plasma membrane rupture, demonstrating that cytokine release can be dissociated from overt cell lysis [48]. ESCRT-III-mediated membrane repair counteracts GSDMD-induced membrane injury and limits both pyroptotic cell death and IL-1β release, suggesting that effective membrane repair may dampen the overall extracellular inflammatory signal rather than selectively favoring cytokine release over lysis-associated markers [46]. In contrast, NINJ1 is required for terminal plasma membrane rupture and the efficient release of large intracellular constituents, including HMGB1 and LDH [45]. Accordingly, a biomarker profile characterized by processed cytokines with relatively limited elevation of HMGB1 or LDH may be consistent with inflammasome/GSDMD activity occurring without terminal membrane rupture, whereas concomitant elevation of DAMPs and cytosolic lysis markers may indicate progression to NINJ1-dependent rupture [45,48]. Testing these relationships in human SCI could determine whether combining caspase-1 and processed cytokines with markers of membrane rupture provides greater information about the cellular consequences of inflammasome activation than caspase-1 measurement alone [45,46].

### 3.3. Biofluid Biomarker

Because activation of the canonical inflammasome produces distinct molecular products, several downstream components of caspase-1 signaling can be detected in CSF, serum, or injured spinal tissue after SCI [10,11]. Caspase-1 activation can be assessed through detection of cleavage products such as p20 or through enzyme activity assays, although cleavage-product detection does not necessarily establish ongoing enzymatic activity, while elevated IL-1β and IL-18 in CSF or plasma serve as indirect indices of inflammasome engagement [49,50]. Cleaved GSDMD fragments and additional cytosolic proteins released through GSDMD-mediated membrane permeabilization also reflect pyroptotic cell death within the injured cord [43,51]. Together, these biofluid markers provide measurable signatures of inflammasome activation that correlate with inflammatory progression, barrier disruption, and secondary tissue loss, and they form the basis for emerging biomarker efforts across neurotrauma and related neuroinflammatory conditions [49,52].

Several technical barriers complicate reliable caspase-1 detection and activity measurement [53]. First, active caspase-1 is highly labile under experimental conditions, with an estimated half-life of approximately 9 min at 37 °C, compared with approximately 8 and 11 h for caspase-3 and caspase-7, respectively [54]. This rapid loss of enzymatic activity suggests that the window for detecting active caspase-1 may be narrow, highlighting the potential importance of preanalytical handling when activity rather than protein abundance is the intended biomarker [54]. Second, antibody-based approaches commonly used for caspase detection are subject to limitations related to antibody specificity and may not, by themselves, establish whether detected caspase species retain enzymatic activity [53,54]. In particular, detection of the p20/p10 cleavage products, historically considered markers of active caspase-1, may instead identify products associated with termination of caspase-1 activity, as the predominant active cellular species include full-length p46 and a transient p33/p10 intermediate [50]. Third, ex vivo inflammasome activation during specimen handling may introduce preanalytical variability into inflammasome-related readouts, including measurements downstream of inflammasome assembly such as caspase-1 [55]. Because caspase-1 activation occurs downstream of inflammasome assembly, such ex vivo activation could plausibly influence caspase-1-related measurements. In unstimulated human whole blood, delayed processing at room temperature resulted in spontaneous ASC-speck formation in monocytes, with a significant increase detectable after 4 h, demonstrating that inflammasome activation can arise during sample storage rather than exclusively reflecting the in vivo inflammatory state [55]. Accordingly, variation in processing delay, temperature, and anticoagulant choice may contribute to preanalytical variability in inflammasome-related measurements and complicate comparisons across studies [55]. Freshly isolated human monocytes exhibit constitutive caspase-1 activity and can release endogenous ATP [56]. These observations raise the possibility that residual viable monocytes could further contribute to ex vivo inflammasome-related activity after specimen collection. Although multiple experimental approaches are available for detecting caspase activity, reliable assessment of biologically active caspase-1 in human biofluids may be complicated by these preanalytical and analytical limitations. More broadly, existing detection methods continue to struggle to keep pace with the growing complexity of known caspase regulatory mechanisms [53].

## 4. Evidence of Caspase-1 Activation After SCI

Evidence for caspase-1 as a biofluid biomarker in SCI exists along a steep gradient. Preclinical data, summarized in Section 3 and Section 5, are extensive and consistent across multiple rodent injury models, but they characterize caspase-1 activation within spinal cord tissue itself, not within a sampled biofluid. This distinction matters because tissue-level mechanistic plausibility does not automatically translate into biofluid detectability. Direct human evidence is limited to a single small cohort study (n = 2), and caspase-1 has not been clinically validated as a biofluid biomarker in SCI (n = 2) [11]. Direct human evidence is limited to a single small cohort study (n = 2), and caspase-1 has not been clinically validated as a biofluid biomarker in SCI. This asymmetry raises a feasibility question that the sections below address directly rather than assume away: CSF collection is invasive and may limit routine serial or widespread sampling, and peripheral blood detection of caspase-1 faces its own barriers, including rapid loss of caspase-1 enzymatic activity under experimental conditions and the difficulty of distinguishing pro-caspase-1 from its cleaved fragments (detailed in Section 3.3).

Whether these constraints are surmountable is, in the authors’ assessment, an open scientific question rather than a closed one. None of the barriers identified are unique to caspase-1 or inherently intractable, and comparable assay and sampling challenges have been overcome for other neuroinflammatory biomarkers. What is currently missing is not evidence against feasibility but the dedicated translational effort, including standardized assays, longitudinal human sampling, and head-to-head comparison with established biomarkers, needed to answer the question directly. This review is intended as a call for that effort rather than a claim that caspase-1’s clinical utility is already established.

### Caspase-1 Levels in Human CSF

In a very small cohort (n = 2) studied by de Rivero Vaccari et al. (2016), patients with acute traumatic SCI (American Spinal Injury Association Impairment Scale [AIS] A-B on admission) demonstrated increased CSF levels of NLRP1, ASC, and caspase-1 within the first 48 h after injury [11]. On immunoblot analysis, the patient with worse neurologic outcome exhibited markedly higher levels of these inflammasome-related proteins than the patient with a better outcome, suggesting a potential association between inflammasome activation and injury severity. To determine whether inflammasome components circulate in vesicle-bound form, the authors isolated exosomes from CSF and found that caspase-1 (including the p20 fragment), ASC, and NLRP1 were enriched in exosomal fractions, particularly in the patient with the worse outcome. These findings suggest that exosome-associated inflammasome signaling is active after acute SCI and that exosomal caspase-1 may have biomarker relevance [11]. Given the sample size of only two patients, these findings preclude any formal statistical inference and should be interpreted as strictly hypothesis-generating. Confirmation in larger, prospectively enrolled cohorts is necessary before any association between inflammasome activation and injury severity can be considered established in humans.

## 5. Caspase-1 Levels in Spinal Tissues (Rodents)

### 5.1. Temporal Dynamics of Caspase-1 in Rodent Spinal Cord Tissue

Following the initial mechanical insult, inflammasome activation contributes to the evolving secondary injury cascade within the spinal cord. In a rat clip-compression (crush) SCI model using female Sprague-Dawley rats, Zheng et al. reported that caspase-1 mRNA expression increased early after injury, peaking at 3 days and remaining elevated at 7 days. IL-1β and IL-18 exhibited similar temporal patterns, consistent with persistent inflammasome activation during the subacute phase [10]. This temporal profile parallels ongoing neuroinflammation and glial activation, suggesting that inflammasome-mediated cytokine release and pyroptotic cell death play a sustained role in amplifying post-traumatic tissue damage [10,57].

### 5.2. Rodent Evidence for NLRP1-Driven Caspase-1 Activation After SCI

Rodent models have provided strong mechanistic evidence for caspase-1-mediated inflammasome activation in SCI, almost entirely at the tissue level rather than in biofluids. In a rat cervical contusion SCI model using adult female Fischer rats, de Rivero Vaccari et al. (2008) demonstrated rapid assembly of the neuronal NLRP1 inflammasome complex, which includes NLRP1 (formerly NALP1), ASC, caspase-1, caspase-11 (the murine ortholog of human caspase-5), and the X-linked inhibitor of apoptosis protein (XIAP) [12,13]. Cleaved caspase-1 was detectable within 15 min of injury and was accompanied by early processing of IL-1β and IL-18. This rapid protein-level cleavage occurs on a substantially faster timescale than the caspase-1 mRNA induction reported by Zheng et al. in a separate clip-compression model, which peaked at 3 days post-injury (Section 5.1) [10]. This apparent discrepancy likely reflects a difference in what is being measured, transcriptional upregulation of caspase-1 mRNA versus post-translational cleavage of pre-existing pro-caspase-1 protein, rather than a true contradiction in injury kinetics, and may also reflect differences between the contusion and clip-compression models used in the respective studies. SCI also induced strong upregulation of caspase-11 and rapid accumulation of ASC within the first hours, whereas NLRP1 levels remained stable and NLRP3 was not detectable in the injured cord [12].

In addition, neutralizing ASC with an antibody reduced caspase-1 activation, decreased IL-1β and IL-18 processing, and improved functional recovery, supporting a causative role for early neuronal inflammasome signaling in the secondary injury cascade. Co-immunoprecipitation studies further revealed dynamic reorganization of the NLRP1 inflammasome complex after SCI, with increased association of ASC with caspase-1 and caspase-11 within 30 min of injury, despite stable NLRP1 levels. Together, those initial findings on the inflammasome in the nervous system suggested that NLRP1 is a rapidly activated inflammasome platform that drives caspase-1 activation and early cytokine processing immediately following mechanical trauma [12]. Importantly, however, these studies quantify inflammasome activation exclusively within spinal cord tissue, leaving unresolved whether caspase-1 or its downstream products are reliably detectable in biofluids or predictive of clinical outcomes after SCI.

### 5.3. NLRP3 Inflammasome Activation and Pyroptosis in Rodent SCI Models

Subsequent mouse and rat studies have extended these findings beyond NLRP1, implicating the NLRP3 inflammasome and pyroptotic cell death in SCI. Across multiple contusion and compression models, SCI induces robust upregulation of NLRP3, ASC, cleaved caspase-1, IL-1β, and IL-18 in spinal cord tissue [58,59,60]. For example, Jiang et al., using female C57BL/6 mice, demonstrated that active (cleaved) caspase-1 remained significantly elevated for up to 72 h after injury, whereas pro-caspase-1 levels did not change, indicating sustained canonical inflammasome activation during the acute–subacute period [58]. Together, these findings support a broader role for inflammasome-driven cytokine maturation and pyroptotic signaling beyond the NLRP1 complex.

Transcript-level studies further corroborate widespread inflammasome activation after SCI. Across several rodent contusion models, SCI induces robust upregulation of NLRP3, ASC, and caspase-1 mRNA within spinal cord tissue. For example, Liang et al. reported marked increases in NLRP3, ASC, and caspase-1 transcripts from 12 h to 7 days after injury in male Sprague-Dawley rats, with these increases partially attenuated by hyperbaric oxygen treatment, suggesting that inflammasome priming is both sustained and therapeutically modifiable [60]. These transcriptional changes complement protein-level data from rodent SCI studies demonstrating sustained elevation of cleaved caspase-1 together with increased IL-1β and IL-18 in the injured cord over the first several days after injury, supporting prolonged canonical inflammasome activation during the acute–subacute period of secondary injury rather than a purely transient post-traumatic response [10,11,58,59].

Notably, nearly all SCI rodent studies assess caspase-1 and inflammasome components exclusively in spinal cord tissue, with minimal evaluation of CSF, serum, or extracellular vesicles. Although these experiments firmly establish caspase-1 activation as a key driver of the secondary injury cascade, they do not determine whether caspase-1 or its downstream products serve as circulating or CSF-based biomarkers. The predominance of tissue-level measurements highlights an important translational limitation: to our knowledge, caspase-1 has been directly evaluated as a measurable clinical biomarker in human SCI in only one small cohort study to date.

### 5.4. Limitations of Rodent Work

Several factors likely contribute to this translational gap. Rodent SCI research has focused predominantly on bilateral thoracic models, whereas the majority of human SCI cases occur at the cervical level and approximately half are incomplete, a mismatch in injury location and severity that may limit the generalizability of preclinical mechanistic findings [61]. Standard post-injury care also differs substantially between experimental rodent studies and human clinical management, particularly regarding analgesia, anticoagulation, and other supportive measures, differences that may alter the post-injury milieu and contribute to divergence between preclinical and clinical inflammatory responses [62]. Circulating biomarker measurements may also be confounded by systemic trauma, as established serum TBI biomarkers such as S100B, NSE, and GFAP that show predictive value in isolated TBI may lose predictive value in the presence of additional extracranial injuries [63]. Consistent with this concern in SCI, acute isolated SCI and polytrauma without neurotrauma have shown differing circulating miRNA patterns, suggesting that trauma context can influence the composition and interpretation of blood-based biomarker signals [64]. These findings, together with evidence that extracranial injury can reduce the specificity of circulating neurotrauma biomarkers, raise the possibility that concomitant systemic injury could complicate interpretation of circulating caspase-1 as a marker of spinal cord-specific inflammasome activity [63,64]. Future multicenter studies evaluating caspase-1 as a human SCI biomarker should standardize sampling time, biospecimen source, analytical methods, and relevant clinical variables, building on existing frameworks for standardized SCI data collection and addressing the methodological heterogeneity already documented in SCI biomarker studies [65,66].

## 6. Therapeutic Inhibition of Caspase-1

### 6.1. Ac-YVAD-cmk

A frequently utilized pharmacologic inhibitor of caspase-1 is Ac-YVAD-cmk, a cell-permeable tetrapeptide chloromethylketone (Ac-Tyr-Val-Ala-Asp-cmk) that targets ICE/caspase-1-like proteases. While widely used as a tool compound, its specificity is incomplete at higher concentrations, and it is therefore best regarded as a caspase-1-preferential rather than a fully selective inhibitor [15,67].

In SCI, early experimental data by Karaoğlan et al. in Wistar rats suggest that post-injury caspase-1 inhibition, achieved via intraperitoneal administration of Ac-YVAD-cmk (1 mg/kg, given 1 min after injury), can attenuate secondary tissue damage [14]. Compared with sham-operated animals, rats subjected to untreated SCI exhibited severe secondary injury marked by diffuse hemorrhage, edema, necrosis, vascular thrombosis, and dense polymorphonuclear leukocyte (PMNL) infiltration within 24 h. After 5 days post-injury, untreated cords demonstrated cavitation, progressive demyelination, axonal degeneration extending from the epicenter, and sustained inflammatory cell burden. In contrast, animals treated with Ac-YVAD-cmk showed significantly reduced PMNL infiltration at 24 h and markedly less cavitation, demyelination, and apoptotic cell death at 5 days, as assessed by histopathology and TUNEL staining [14]. These structural improvements were accompanied by superior functional outcomes, with significantly higher inclined-plane and motor scores compared with untreated trauma controls, indicating that early caspase-1 inhibition mitigates inflammatory-mediated tissue destruction and supports neurological recovery [14].

Ac-YVAD-cmk has also been evaluated in a zebrafish spinal cord injury model, in which larvae were exposed to the inhibitor in fish water at 50 μM. Pharmacologic caspase-1 inhibition altered IL-1β-associated inflammatory responses and axonal regeneration, with effects that varied according to the timing of treatment [68].

### 6.2. VX-765 (Belnacasan)

VX-765 (belnacasan) is an orally bioavailable prodrug that is rapidly converted in vivo to the active metabolite VRT-043198, a highly potent and selective inhibitor of the caspase-1 subfamily [11,49,50]. In biochemical and cellular assays, VRT-043198 exhibits approximately 100- to 10,000-fold selectivity for caspase-1 over other caspases and suppresses inflammasome-dependent IL-1β and IL-18 release in human peripheral blood mononuclear cells and whole blood, while largely sparing non-inflammasome cytokines [69].

More recently, VX-765 has been evaluated in experimental SCI. In a T9 contusion model using C57BL/6 female mice, Chen et al. demonstrated that VX-765 administered systemically by intraperitoneal injection (100 mg/kg immediately after injury and once daily for 7 days) significantly reduced cleaved caspase-1 and downstream IL-1β and IL-18 levels in injured spinal cord tissue, while shifting myeloid and T-cell populations toward an anti-inflammatory phenotype [16]. These molecular effects were accompanied by a smaller lesion size, greater preservation of myelinated white matter and ventral horn motor neurons, and significantly improved locomotor recovery on the Basso Mouse Scale beginning at 21 days post-injury, supporting caspase-1 inhibition as a therapeutic strategy in SCI [16].

At the signaling level, immunoblotting further showed that SCI markedly increased the cleaved, active forms of both caspase-1 and caspase-3 without significantly altering pro-caspase expression, whereas VX-765 treatment robustly suppressed activation of both proteases [70]. These findings position caspase-1 upstream of canonical inflammasome signaling and downstream apoptotic cascades following traumatic SCI. Collectively, these studies firmly establish caspase-1 as a mechanistic driver of secondary injury in experimental SCI. While these results support caspase-1 inhibition as a promising therapeutic strategy, they should not be interpreted as validating caspase-1’s utility as a biomarker; a protein can be a viable therapeutic target without being a reliable diagnostic or prognostic biofluid marker, and the two questions require independent evidentiary support. These experimental results further support caspase-1 as a therapeutically relevant target in SCI. However, the absence of validated biomarkers of inflammasome activation in human SCI limits the ability to identify patients most likely to benefit from such interventions or to monitor treatment response in clinical trials.

## 7. Caspase-1 in the Broader SCI Biomarker Landscape

Despite the emergence of numerous candidate biomarkers, biomarker research in SCI remains largely dominated by preclinical studies. Markers of astroglial injury include calcium binding protein S100 beta (S100β) and glial fibrillary acidic protein (GFAP). Candidate markers of neuronal, axonal, or myelin injury encompass ubiquitin C-terminal hydrolase-L1 [UCHL-1], neuron-specific enolase [NSE], myelin basic protein (MBP), neurofilament protein-H and L [NF-H, NfL], and microtubule-associated 2 [MAP-2]. αII-spectrin breakdown products (SBDP150/SBDP145/SBDP120) reflect cytoskeletal proteolysis while interleukin-6 (IL-6) and interleukin-8 (IL-8) represent inflammatory biomarkers [71,72]. Of these, SBDP150/145, UCH-L1, GFAP, and S100β have been measured at significantly higher concentrations in the tissue or CSF of rat models with SCI, as well as prospectively in SCI patients, compared to healthy participants [72,73]. UCHL-L1, S100β, and GFAP were also higher in patients with AIS grade A injuries, suggesting a relationship between biomarker levels and injury severity [73]. Wang et al. identified an additional twelve proteins as candidate SCI biomarkers using rat models; of these, Transferrin, Cathepsin D, Triose-phosphate isomerase 1 (TPI-1), and Phosphoprotein enriched in astrocytes 15 (PEA15) were verified as elevated in human CSF samples from SCI patients [71].

Against this background, caspase-1 should be considered complementary to, rather than inherently superior to, established injury biomarkers. Markers such as GFAP, UCH-L1, NfL, and SBDPs predominantly report astroglial, neuronal, axonal, or cytoskeletal injury, whereas cleaved caspase-1 or caspase-1 enzymatic activity could more directly reflect canonical inflammasome engagement. However, this proposed mechanistic specificity has not been validated in human SCI. Assays must distinguish pro-caspase-1, cleaved caspase-1 fragments such as p20, and enzymatic activity, while circulating measurements may also be affected by systemic trauma and inflammation. Head-to-head longitudinal studies comparing caspase-1 with established biomarker panels are needed to determine whether it provides additional diagnostic, prognostic, or pharmacodynamic value.

## 8. Translational Barriers

Despite strong preclinical support for caspase-1 inhibition in SCI, several challenges have impeded translation to human studies. Clinical data are virtually absent, with, to our knowledge, no early-phase trials evaluating caspase-1 inhibitors, NLRP3 blockers, or downstream IL-1β/IL-18 antagonists in acute SCI. As a result, the optimal therapeutic window remains uncertain. Although rodent data demonstrate robust inflammasome activation within the first 72 h after injury, it is unclear whether this timeline applies to humans or whether ultra-acute intervention is required. In addition, systemic inflammasome inhibition may produce off-target immunosuppression, increasing susceptibility to infection, or interfering with necessary inflammatory repair processes, given the established role of IL-1β and IL-18 in host defense against bacterial infection. Clinical evidence for this concern is strongest for therapies acting downstream of caspase-1: in randomized trials of patients with rheumatoid arthritis, the IL-1 receptor antagonist anakinra was associated with a numerically higher incidence of serious infections than placebo, with a statistically significant increase in risk observed at doses >100 mg/day, while broader clinical experience with IL-1-targeted therapies has also identified infectious adverse events [74,75]. Whether caspase-1 inhibition itself carries comparable infectious risk is less clear. In a Phase IIa trial of VX-765, 72.9% of treated participants experienced at least one treatment-emergent adverse event compared with 83.3% receiving placebo, although serious treatment-emergent adverse events occurred in 6.3% of VX-765-treated participants compared with 0% of placebo-treated participants [76]. These limited data do not establish whether caspase-1 inhibition carries a comparable infectious risk; differences in mechanism, trial size, duration, and patient population, together with the paucity of caspase-1 inhibitor trials, preclude firm conclusions. If clinically validated, serial measurement of caspase-1 activity could be explored as a pharmacodynamic marker in future trials, potentially informing the degree and duration of inflammasome suppression while being interpreted alongside infection-relevant immune parameters. Finally, validated biomarkers of inflammasome activation are lacking. Candidate measures—including CSF IL-1β, IL-18, ASC specks, and cleaved GSDMD fragments—have not been standardized, longitudinally evaluated, or linked to neurological outcomes in humans. These limitations complicate dose selection, patient stratification, and trial design, underscoring the need for dedicated translational studies.

## 9. Conclusions

Caspase-1 is a central mechanistic node at the intersection of inflammation and programmed cell death in SCI, integrating upstream danger signals with IL-1β/IL-18 production and pyroptosis execution. Across multiple rodent models, pharmacologic suppression of caspase-1 consistently improves outcomes, including reduced lesion pathology, attenuated inflammatory signaling, and improved locomotor recovery [12,14]. Despite decades of promising preclinical work, the lack of human biomarker studies, translational models, and early-phase clinical trials continues to limit progress. Therefore, advancing this field will require coordinated efforts to map human inflammasome kinetics, establish measurable biomarkers, test selective inhibitors, and determine whether the robust benefits seen in animal models can be replicated in patients with acute SCI.

## Data Availability

No new data were created or analyzed in this study. Data sharing is not applicable to this article.

## Abbreviations

The following abbreviations are used in this manuscript:

Ac-YVAD-cmk	Acetyl-Tyr-Val-Ala-Asp chloromethyl ketone
AIM2	Absent in melanoma 2
AIS	American Spinal Injury Association Impairment Scale
ASC	Apoptosis-associated speck-like protein containing a caspase activation and recruitment domain
ATP	Adenosine triphosphate
BBB	Blood–brain barrier
BSCB	Blood–spinal cord barrier
C1q	Complement component 1q
CSF	Cerebrospinal fluid
DAMP	Damage-associated molecular pattern
ESCRT	Endosomal sorting complex required for transport
ESCRT-III	Endosomal sorting complex required for transport III
GFAP	Glial fibrillary acidic protein
GSDMD	Gasdermin D
HMGB1	High-mobility group box 1
ICE	Interleukin-1β-converting enzyme
IL	Interleukin
IL-1R	Interleukin-1 receptor
LDH	Lactate dehydrogenase
MAP2	Microtubule-associated protein 2
MAVS	Mitochondrial antiviral-signaling protein
MBP	Myelin basic protein
MDP	Muramyl dipeptide
mRNA	Messenger RNA
NEK7	NIMA-related kinase 7
NF-H	Neurofilament heavy chain
NF-κB	Nuclear factor kappa B
NfL	Neurofilament light chain
N-GSDMD	N-terminal gasdermin D fragment
NINJ1	Nerve injury-induced protein 1
NLRP1	NLR family pyrin domain containing 1
NLRP3	NLR family pyrin domain containing 3
NOD2	Nucleotide-binding oligomerization domain-containing protein 2
NSE	Neuron-specific enolase
PAMP	Pathogen-associated molecular pattern
PEA15	Phosphoprotein enriched in astrocytes 15
PMNL	Polymorphonuclear leukocyte
PMR	Plasma-membrane rupture
ROS	Reactive oxygen species
S100B	S100 calcium-binding protein B
SBDP	αII-spectrin breakdown product
SCI	Spinal cord injury
TLR	Toll-like receptor
TNF	Tumor necrosis factor
TNFR	Tumor necrosis factor receptor
TPI1	Triosephosphate isomerase 1
TUNEL	Terminal deoxynucleotidyl transferase dUTP nick-end labeling
UCH-L1	Ubiquitin C-terminal hydrolase L1
XIAP	X-linked inhibitor of apoptosis protein
